# Algorithm for multi-curve-fitting with shared parameters and a possible application in evoked compound action potential measurements

**DOI:** 10.1186/1475-925X-5-13

**Published:** 2006-02-22

**Authors:** Philipp Spitzer, Clemens Zierhofer, Erwin Hochmair

**Affiliations:** 1 University of Innsbruck, Institute of Applied Physics, Technikerstrasse 25, 6020 Innsbruck, Austria; 2 Christian Doppler Laboratory for Active Implantable Systems, University of Innsbruck, Institute of Applied Physics, Technikerstrasse 25, 6020 Innsbruck, Austria

## Abstract

**Background:**

Experimental results are commonly fitted by determining parameter values of suitable mathematical expressions. In case a relation exists between different data sets, the accuracy of the parameters obtained can be increased by incorporating this relationship in the fitting process instead of fitting the recordings separately.

**Methods:**

An algorithm to fit multiple measured curves simultaneously was developed. The method accounts for parameters that are *shared *by some curves. It can be applied to either linear or nonlinear equations. Simulated noisy "measurement results" were created to compare the introduced method to the "straight forward" way of fitting the curves separately.

**Results:**

The analysis of the simulated measurements confirm, that the introduced method yields more accurate parameters compared to the ones gained by fitting the measurements separately. Therefore it needs more computer time. As an example, the new fitting algorithm is applied to the measurements of the evoked compound action potentials (ECAP) of the auditory nerve: This leads to promising ideas to reduce artefacts generated by the measuring process.

**Conclusion:**

The introduced fitting algorithm uses the relationship between multiple measurement results to increase the accuracy of the parameters. Its application in the field of ECAP measurements is promising and should be further investigated.

## Background

It's very common to analyse a system by making measurements and trying to fit a mathematical equation (i.e. the *model *of the system) to the results. That way the system is described by the fitted parameters. If the system is complex and the equation has many (*M*) parameters *a*_1 _to *a*_*M *_(described by the vector ***a***), receiving usable values from only one measurement/fit is difficult. In this case, one possibility is to make more than one (*N*) measurement and alter some of the test-conditions which should appear in one or more parameters of the fittings. If the results of these measurements were fitted separately, the situation would not improve much. Here an algorithm is introduced that fits those *N *measurements simultaneously to *N *equations which may be (but need not) different and may share some of the parameters *a*_*m*_.

## Example

**Figure 1:**
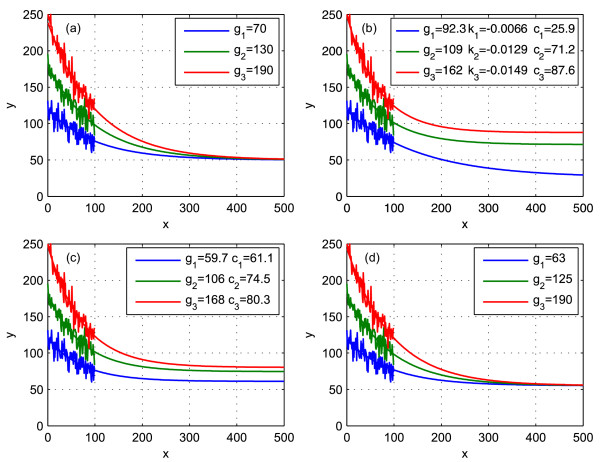
**Example task**. Three curves of the form *y *= *g*_*i *_*e*^*kx *^+ *c *were created (a) with *k *= -0.01 and *c *= 50. Then the range between *x *= 0 and *x *= 99 was taken and noise was added. The task for the fit methods was to extract the original parameters using only this noisy part. It was done by (b) fitting each curve separately with *y *=  + *c*_*i*_, (c) taking the introduced fit method with shared *k *(*y *= *g*_*i*_*e*^*kx *^+ *c*_*i *_where *k *was determined to be -0.0137) and (d) with shared *k and c *(*y *= *g*_*i*_*e*^*kx *^+ *c *where *k *and *c *resulted in -0.0107 and 55.2 respectively).

We have *N *= 3 curves described by the equations *y*(*x*) = *g*_*i *_*e*^-*kx *^+ *c *(*i *= 1 ... 3) which are degraded by added normally distributed noise to simulate the measurement process (left part of figure 1(a)). The task is to retrieve the parameters used to create the curves by only using the degraded values for 0 ≤ *x *< 100. When the three curves are fitted separately, one obtains three values of each parameter *g*, *k *and *c *(figure 1(b)).

The fit method we are introducing here is able to fit these curves simultaneously and takes into account that the parameters *k *and *c *are *shared*. Therefore it returns only one value for *k *and one value for *c *(figure 1(d)). The following equations were used for fitting:

*y*_3_(*x*) = g_3 _*e*^-*kx *^+ *c *    (1)

*y*_2_(*x*) = *g*_2 _*e*^-*kx *^+ *c *    (2)

*y*_1_(*x*) = *g*_1 _*e*^-*kx *^+ *c *    (3)

In this example, the vector of the fit-parameters ***a ***was assigned as follows:

*a*_1 _= *k * *a*_2 _= *c * *a*_3 _= *g*_1 _ *a*_4 _= *g*_2   _*a*_5 _= *g*_3_

Figure 1(c) shows the result when using this algorithm with shared *k *only.

## Principle of the algorithm

The basic idea is the following: When fitting *one *curve to *one *equation, the *goodness of the fit parameter χ*^2 ^(weighted sum of the quadratic deviations of the values – definition follows) is minimised. To fit *N *curves to *N *equations simultaneously, the *sum *of the individual *χ*^2 ^values has to be minimised. Definitions:

• *N *... Number of equations/curves. Each curve is represented by one equation.

• *A*(*n*) ... Number of the nodes belonging to the *n*th measured curve.

• (*x*_*ni*_, *y*_*ni*_, ) ... node number *i *of the *n*th curve (*i *= 1 ... *A*(*n*)). *σ*^2 ^is the uncertainty and can be set to 1 if it is identical for all nodes of all measurements.

• *M *... Total number of parameters that should be determined with the fit.

• ***a ***= (*a*_1_, ..., *a*_*M*_) ... *M *element vector of the fit-parameters *a*_1 _to *a*_*M*_.

• *y*_*n*_(*x*, ***a***) ... *n*th equation. It can use *all *fit parameters ***a***, but there are three possibilities the *n*th equation could use every single parameter *a*_*m*_:

1. The equation is independent of *a*_*m*_. Then the (in the following needed) partial derivation of the equation according to this parameter *a*_*m *_is zero.

2. *a*_*m *_is a "normal" fit-parameter, that should be optimised.

3. The equation depends on *a*_*m*_, but should not partake in the optimisation. This is done by setting the partial derivation for this parameter to zero.

The total sum of the goodness of the fit parameter *χ*^2 ^is defined as sum of the goodness of the fit parameters  of each curve. The bigger *χ*^2^, the worse the fit:





## Method for linear functions

If all equations *y*_*n*_(*x*, ***a***) are linear in the *parameters ****a***, we can use the least square fit method [1] to minimise the total *χ*^2^. In this case, the equations *y*_*n*_(*x*, ***a***) have to be of the following form:



*f*_*nm*_(*x*) are arbitrary functions. Note that a polynomial function is a special case of this where *f*_*nm*_(*x*) has e.g. the form *f*_*nm*_(*x*) = *x*^*m*^. To get the values for ***a***, the value of *χ*^2 ^is minimised by setting the derivations according to each parameter *a*_*k *_to zero:









In this way one gets *M *linear equations (*k *= 1 ... *M*) to determine the *M *entries of ***a***. Equation 10 may look complicated, but all values except *a*_*m *_are explicitly known. The equation can be solved by the method of determinants.

The advantages of the least square method over all non-linear methods are very fast processing, final results in one step and no need to specify starting parameters.

## Method for nonlinear functions

If the equations *y*_*n*_(*x*, ***a***) depend on some of the parameters ***a ***in a non-linear way, the requirement to use the least square fit method is not met. In this case, one of the fastest methods to minimise *χ*^2 ^is the Marquardt-Method [2], an iterative numerical process. Here it is adapted to fit *N *curves simultaneously. We use the Marquardt method here, because some of the ideas that are described later require non-linear fitting. The following description can be considered a recipe. For the mathematical background of the Marquardt-Fit-Method, see [1,2].

1. Calculate *χ*^2 ^with suitable starting parameters ***a ***with equations (4) and (5).

2. Set *λ *to 10^-3^.*λ *is a constant factor that controls whether the Marquardt-Fit-Method should behave more as a gradient search fit method (*λ *≪ 1) or an expansion fit method (*λ *≫ 1).

3. Calculate the vector ***δa***, which has the same size as ***a ***(namely *M *elements) and describes the suggested correction to ***a ***as follows:

(a) The *M *element vector ***β ***represents the first partial derivations of *χ*^2 ^to the fit parameters described by ***a***:



(b) The *M *× *M *Matrix  describes the second partial derivations to the fit parameters *a*_*k *_and *a*_*m *_(*k *and *m *vary from 1 to *M *respectively):



(c) Calculate the matrix ***α ***by multiplying each diagonal element from  by (1 + *λ*).

(d) Determine the inverse matrix ***ε ***of ***α***.

(e) Calculate ***δa ***as follows:



4. Derive new trial-fit-parameters ***a' ***from the "old" ones ***a***:

***a' ***= ***a ***+ ***δa ***    (14)

5. Determine *χ'*^2 ^with the trial-fit-parameters ***a' ***using equations (4) and (5 and (5)).

6. If *χ'*^2 ^≥ *χ*^2 ^(i.e. there is no improvement with the trial-fit-parameters): Substitute *λ *with 10·*λ*, keep ***a ***unchanged, and continue with step 3.

7. If *χ'*^2 ^<*χ*^2 ^(i.e. the new parameters are better): Substitute *λ *with *λ*/10, and ***a ***with ***a' ***and continue with step 3.

This iterative calculation may be stopped when one of the three following conditions is met:

• *χ*^2 ^falls below a certain predefined threshold.

• The difference between *χ*^2 ^and *χ'*^2 ^decreases to less than a specified value.

• The count of the calculation loops exceeds a maximum number.

Note: Using this method, one has to treat "constant" parameters (parameters that do not participate in the fitting process) in a special way (and not within ***a ***having derivations of zero), because otherwise their value may be biassed to minimise *χ*^2^. This could be done by an extra parameter for *y*_*n*_(*x*, ***a***) (e.g. called ***b***) or by incorporating the constant parameters within the functions *y*_*n*_(*x*, ***a***).

## Comparsion to single fits

**Figure 2:**
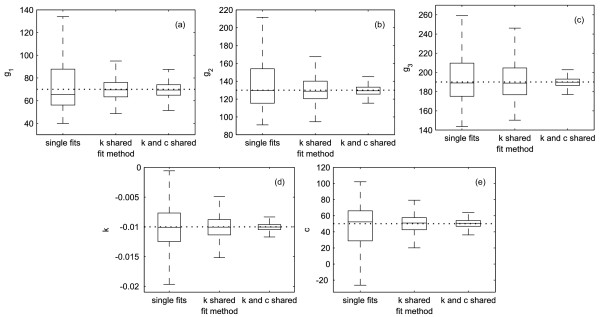
**Results of the example task**. Results of the example task shown in figure 1: The task was done 1000 times with other noise signals. The distribution of the obtained parameters is shown here as box and whisker plots. The dashed lines show the template values. (a) distribution of *g*_1_. (b) distribution of *g*_2_. (c) distribution of *g*_3_. (d) distribution of *k*. (e) distribution of *c*.

The benefit of parameter-sharing is that *all *retrieved values (not only the shared ones) are more precise compared to fitting them separately. To verify this, 1000 fits of the simulated "measurement" from the example above had been made with the following three methods: single fits, simultaneous fits with shared *k *and simultaneous fits with shared *k *and *c*. For every fit, a "new" noise was used. The distributions of the parameters obtained with the three different methods are shown in figure 2: The smallest deviation from the real parameters were obtained by the fits, where *k and c *were shared.

## Use with cochlear implant ECAP measurements

Cochlear implants are medical devices that enable deaf people to perceive hearing impressions by electrically stimulating the auditory nerve [3]. The stimulations occur through a multichannel electrode that is placed directly into the cochlea and has contacts on different positions. This way, up to 100% speech recognition can be achieved [4].

Modern cochlear implants can record the evoked compound action potential (ECAP) of the auditory nerve. This is done by measuring the voltage at one of the contacts of the multichannel electrode after a stimulation pulse that evokes the action potentials. The recordings obtained show the sum of the ECAP and exponential decays resulting from residual charges (arising from capacitive components on the implant itself or from the electrode-electrolyte transition). There are several well established recording methods to eliminate or reduce this so called *artefact *to obtain the pure ECAP: Alternating stimulation [5], masker probe methods [6,7], triphasic stimulation [8] and scaled template methods [9]. However, each method has its limitations: Some of them rely on the linearity of the system (alternating stimulation), others need up to between nine to sixteen times more measurements for one result (masker probe methods).

Another issue is the influence of the used measurement system itself, which may be seen on the measured curves as offsets, drifts, additional exponential decays, ... (called *system effects *here).

The introduced method can be used as a tool to help separate the ECAP signal from these undesired components. One way to do so is to fit just the artefact (without the ECAP) along with the system effects and subtract this fit from the measurements. This can be done with sub-threshold measurements, recovery measurements, or in regions, without an ECAP. Another possibility is to combine this method with an artefact cancellation method mentioned above, for example to eliminate system effects.

The algorithm needs sequences with different conditions to gain advantage over single-curve-fits. In practical use this is no disadvantage, because usually two special sequences are measured: The *amplitude growth sequence *and the *recovery sequence*. The amplitude growth sequence raises the stimulation pulse amplitude from zero to the maximum comfortable loudness (MCL) level. The recovery sequence sends two MCL level pulses before the measurement – if the second pulse lies within the auditory nerve recovery time of the first pulse (about < 1 ms), the ECAP vanishes.

## Example ECAP measurement

**Figure 3:**
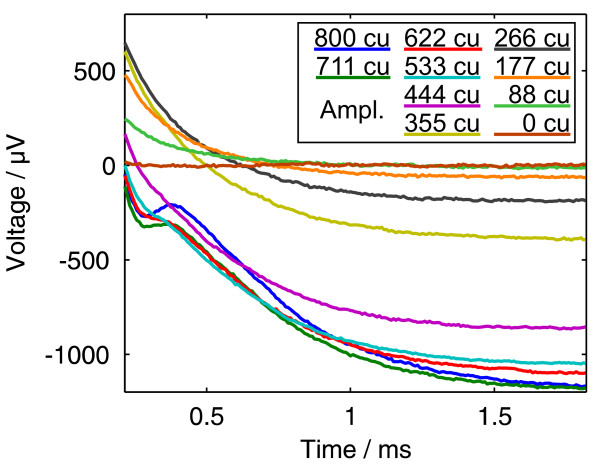
**Amplitude growth measurement result**. Data from an Amplitude Growth measurement with the MedEl PULSARCI^100 ^cochlear implant. For each curve, 50 cathodic/anodic pulses were averaged. The pulses had an amplitude between 0 and 800 cu (1 cu ≈ 1 *μ*A). Their begin is the time origin.

Figure 3 shows the results of an ECAP measurement using the amplitude growth sequence with ten amplitudes between 0 and 800 cu (current units, 1 cu corresponds nominally to 1 *μ*A). It was recorded with the MedEl PULSARCI^100 ^cochlear implant and the software ArtResearch. Biphasic pulses beginning with the cathodic phase were used. For each of the ten curves, 50 recordings were taken and averaged. We intentionally chose an ECAP measurement with a large artefact and with different offset voltages for each curve to demonstrate the power of the algorithm.

The following equation was used as "model" of the artefact plus offset for each curve. We assumed that the artefact can be described by two exponentially decreasing terms and one offset parameter.

*y*(*t*) = *fe*^-*t*/*τ *^+ *ge*^-*t*/*T *^+ *c *    (15)

The final offset c and the amplitudes *f *and *g *are different for each curve, but the time constants *τ *and *T *are assumed to be global, so that the following ***a ***was used. It contains 32 parameters.

***a ***= (*τ*, *T*, *f*_1_, *g*_1_, *c*_1_, *f*_2_, *g*_2_, *c*_2_, ..., *f*_10_, *g*_10_, *c*_10_)     (16)

As mentioned above, several ideas exist to take advantage of the algorithm. Three of them will be described in the following subsections:

### Fit of the regions without an ECAP signal

The first possibility is to exclude the regions of the curves where an ECAP signal is expected from the fit. In this example, we included the whole range of the five measurements from 0 to 355 cu stimulation amplitude that are sub-threshold (figure 3), and the right part of the other five measurements, where the ECAP signal (duration of about 1 ms [10]) has vanished.

**Figure 4:**
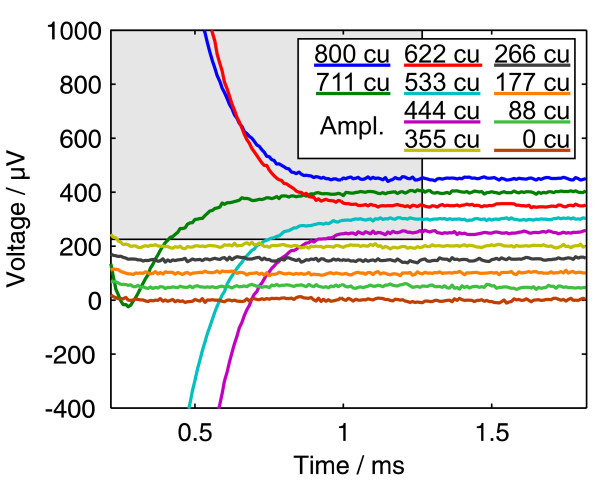
**Data minus fit**. This figure shows the same data as in figure 3 but with a fit subtracted. The selected region was excluded from the fit. The lines were plotted with an offset of 50 *μ*V each. Details in text.

The run time of the algorithm was about one minute (interpreted Matlab™ code with no attempts to speed it up), calculating the fitted values for the 32 parameters. As expected, straight lines were obtained for the fitted regions when subtracting the fit from the original data, indicating a tight fit (figure 4). The returned values for the time constants were: *τ *= 322 *μ*s and *T *= 138 *μ*s. What we could not expect was that the fit was good for the regions that did not partake in the fitting process, because those regions of the fit where *g*_6 _to *g*_10 _(the factors for the fast exponential function) could be determined properly were excluded from the fit as described before.

These patient specific time constants describe mainly the electrical properties of the electrode-electrolyte interface [11] and the geometry, and could be taken as diagnostic parameters along with the ECAP amplitude or the residual voltage measured in telemetry measurements.

**Figure 5:**
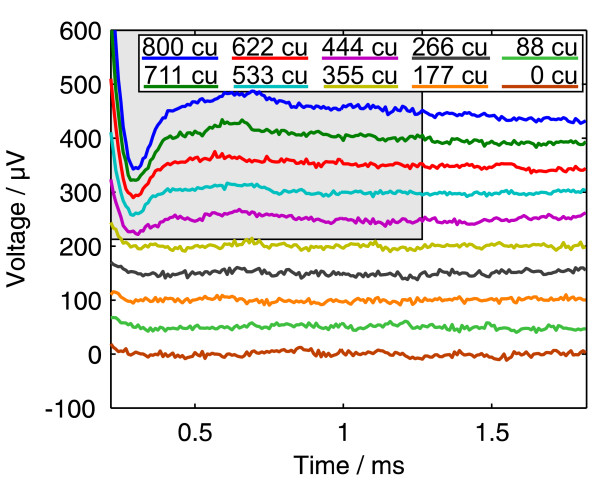
**Additional fit**. Same as figure 4 with additional fit to obtain values for the parameters that describe the fit within the region that was excluded for the first fit. The lines were plotted with an offset of 50 *μ*V each. Details in text.

If the ECAP signal is small compared to the amplitude of the exponential decay, a good guess of *f*_6 _to *f*_10 _and *g*_6 _to *g*_10 _would yield a further fit over *all *regions where the other coefficients are kept constant. We have done so in keeping *τ *and *T *constant whereas the other parameters were calculated for the five "high current" curves. The result of this is shown in figure 5.

A remark on the starting parameters: The method we used to get the starting parameters was to fit the curves with a simplified model having only one exponential term (with shared time constant) and taking into account only the right halves of the curves. In this way we obtained values for the long time constant *τ*, *f*_*i *_and *c*_*i*_. We then subtracted the fits of this simplified model from the curves and fitted again, using only one exponential term with a shared time constant *T *and taking into account the whole curve. After that we got starting values for *g*_*i *_and *T*. These additional fits needed only a few seconds calculation time.

### Using *σ*^2 ^to fit over all regions

Instead of first excluding the parts of the curves with an ECAP signal and then fitting over all regions where some of the previously determined parameters are kept constant, we could initially fit over all regions and use the uncertainty parameter *σ*^2 ^(see section "Principle of the algorithm") to characterise regions where the ECAP signal is expected: These regions should receive a high *σ*^2 ^value compared with the *σ*^2 ^value from the other regions, so that the fitting algorithm doesn't punish the ECAP caused deviations with a high contribution to the *χ*^2 ^value.

The more of the following conditions are satisfied, the better the results from this approach:

• The ECAP amplitude is small compared to the amplitude of the artefact.

• There is a region before and after the ECAP-part of the curve that is described by the artefact model used.

• The ECAP signal is dc-free, i.e. the integral over the ECAP signal (without any artefact) is very small.

• The artefact time constants are larger than the ECAP time constants.

**Figure 6:**
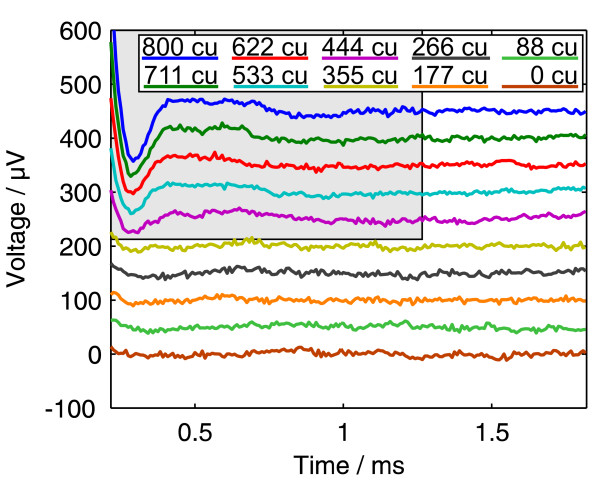
**Influence of *σ*^2^**. Data from figure 3 minus fit where the *σ*^2 ^value of the selected area was 10 instead of 1. The lines were plotted with an offset of 50 *μ*V each.

Figure 6 shows the results of this variant when using *σ*^2 ^= 10 instead of 1 within the selected areas where the ECAP signal is expected. The retrieved values for *τ *and *T *were 328 *μ*s and 176 *μ*s.

### Using an ECAP model to fit over all regions

A further approach is to model not only the artefact, but the ECAP signal too. [12] would be an example for a model that could be used. This model takes into account the double-peak shape of some ECAPs as well. Doing so is part of current investigation and may be published in the future.

## Conclusion

The introduced fit-algorithm uses the additional information of the relation between measured curves to retrieve more accurate parameters compared to the parameters extracted from single fits. In the case of ECAP measurements the algorithm could be used as (additional) artefact cancellation method with the following benefits:

• There are no additional measurements necessary to apply this method.

• The method can be used in combination with other artefact cancellation methods.

• It can take into account implant specific system effects.

• Physiological or implant specific parameters like time constants or the artefact amplitude are gained as values that can be used for diagnostic purposes.

• No additional noise is added.

• It is very flexible because of the possibilities to judge different regions of a curve and different curves in special ways with the *σ*^2 ^parameter (e.g. curves with zero pulse amplitude fit some of the parameters very accurately), leave out data which should not be included in the fit (e.g. holes where the ECAP is expected), use different equations for different curves (e.g. combining results of amplitude growth and recovery sequences), and exclude parameters of some curves from the fit process (e.g. time constants in curves with ECAP).

• Constant parameters (time constant, offset, ...) can be reused (at least as starting parameters) to save calculation time.

The drawbacks are that the calculation can be time-consuming (especially with many fit parameters) and that there have to be suitable starting parameters. The first issue can be improved by reducing the resolution of the curves to receive a rough result, and optimising the stop condition for the calculation loop to fit the problem. The second issue can be dealt with by reusing parameters from earlier runs, or by determining them by doing rough pre-fits with less complicated equations.

Further investigation is necessary to develop a final method based on the introduced ideas. ECAP signals obtained with this method should be compared in size and shape with results of traditional artefact cancelation methods.

## Authors' contributions

PS developed the basic method and wrote the draft of the publication. It was discussed, criticised, and partly rewritten to clarify the presentation together with EH and CZ. They had the idea of the comparison with the straight forward approach of fitting the curves separately by doing simulated measurements.
